# Fermi Edge of Semimetallic Borophane Sheets and its Reduction by a Porous Structure

**DOI:** 10.1021/acs.jpclett.4c01869

**Published:** 2024-09-06

**Authors:** Xiaoni Zhang, Masashige Miyamoto, Mei Yuan, Yuki Tsujikawa, Kazuki Yamaguchi, Masafumi Horio, Kenichi Ozawa, Kunio Yubuta, Takahiro Kondo, Iwao Matsuda

**Affiliations:** † Institute for Solid State Physics (ISSP), The University of Tokyo, Kashiwa, Chiba 277-8581, Japan; ‡ Institute of Pure and Applied Sciences, University of Tsukuba, Tsukuba, Ibaraki 305-8573, Japan; ¶ Institute of Materials Structure Science, KEK, Ibaraki 305-0801, Japan; § Faculty of Engineering, Kyushu University, Fukuoka, Fukuoka 819-0395, Japan

## Abstract

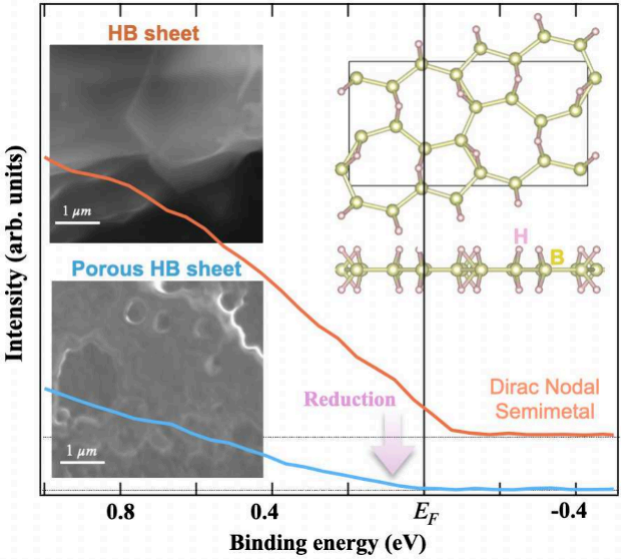

Theoretically predicted materials are often synthesized in low yields, and unexpected relationships are often encountered between the target materials and byproducts. Recently, two-dimensional boron materials proposed on the basis of model simulations and first principles calculations and possessing abundant atomic structures have attracted considerable interest. Borophane or the hydrogen boride (HB) sheet has been predicted to be the Dirac nodal semimetal when it has a boron network of nonsymmorphic symmetry. Upgrading the standard method, we fabricated freestanding HB sheets possessing either an apparent Fermi edge, reduced spectral weight, or a Fermi-level energy gap, as confirmed by using microbeam photoemission spectroscopy. The gapless electronic structures were correlated with terminal B–H bonds at the sheet edges, indicating the electronic modification of the porous structure as directly microscopically observed. The gapped or insulating sheet was fabricated *via* oxidation. This research provides methods for regulating the structural morphology and electronic states of HB sheets during synthesis.

Because of their wide range of physicochemical properties, two-dimensional (2D) boron-based materials have garnered considerable attention,^1−10^ particularly borophene^8−11^ and its hydrogenated counterpart, borophane or hydrogen boride (HB) sheets.^12−20^ Stable freestanding HB sheets have been fabricated by chemical method and sharply contrast with borophene layers,^13,17^ which require crystal substrates and ultrahigh vacuum (UHV) conditions.^8,11^ HB sheets possess different boron atomic arrangements, including honeycomb- and β_12_-type lattice structures.^17,18^ Individual HB layers possess unique electronic states, and HB sheets possessing a boron atomic network comprising five- and seven-membered rings (pentagons and heptagons),^12−15^ herein referred to as 5,7-HB, have been proposed as being Dirac nodal semimetallic. Figure 1a shows the atomic structural models of 5,7-HB possessing different hydrogen atomic arrangements, α_1_ and α_2_. In HB sheet structures, boron and hydrogen atoms are bonded in a three-center–two-electron (3c–2e) configuration. The *Z*_2_ topology was theoretically predicted based on the nonsymmorphic symmetry group of 5,7-HB.^12,19,20^ Since 5,7-HB sheets have been synthesized by chemically exfoliating metal borides via ion exchange,^13,14,17^ there is growing scientific interest in investigating the electronic state topology of boron polymorphs.^13^

**Figure 1:**
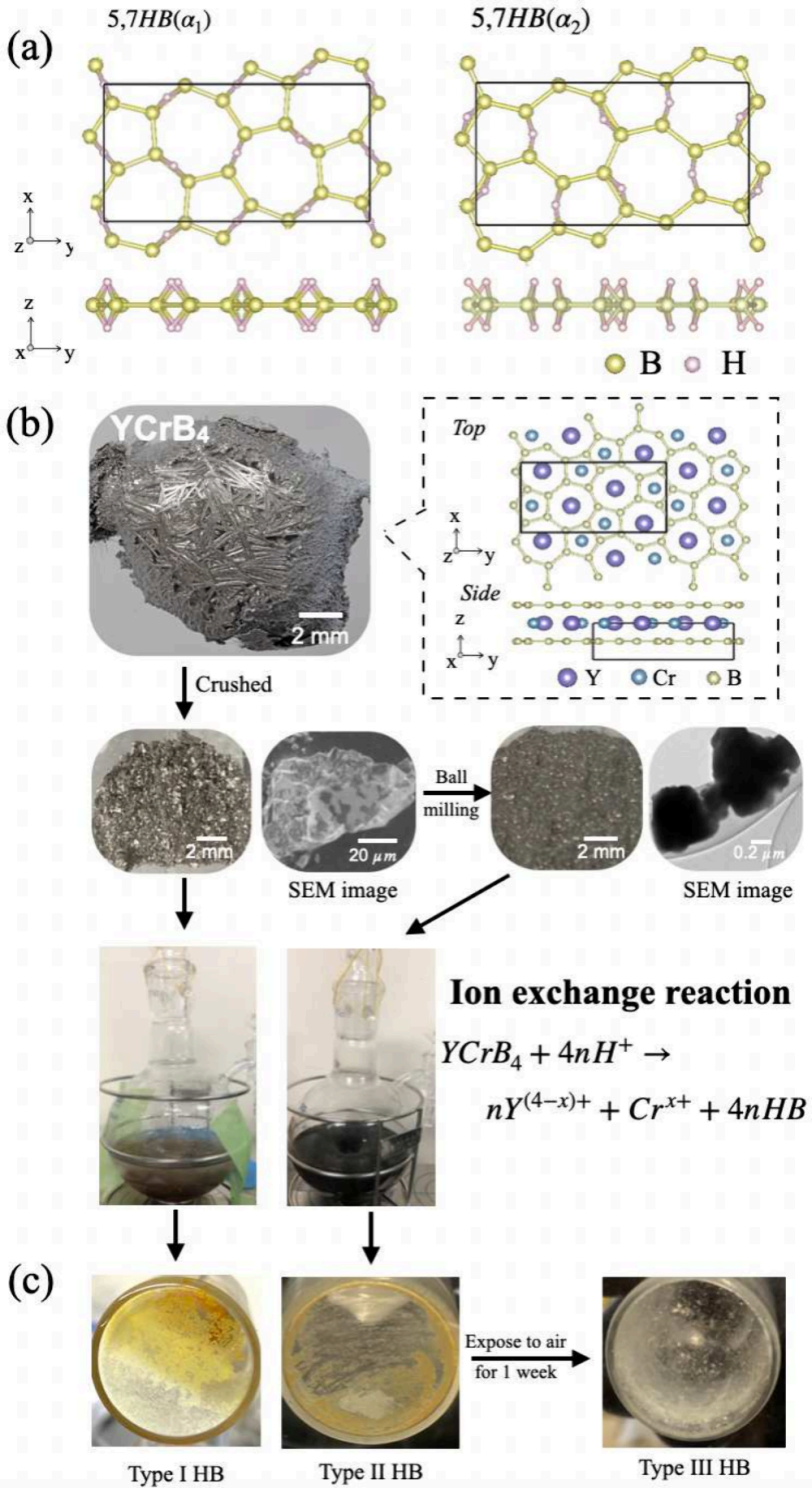
Synthesis of three types of 5,7-HB sheets. (a) Atomic structures of α_1_- and α_2_-5,7-HB. (b) Synthesis of three types of 5,7-HB sheets derived from YCrB_4_ crystals; the atomic structure of YCrB_4_ is depicted inside the dashed line. Crushed (left) and ball-milled (right) YCrB_4_ crystals are mixed with a cation exchange resin to initiate the ion exchange. The scanning electron microscopy (SEM) image confirms that the ball-milled YCrB_4_ crystals are smaller than 10 μm. (c) As-synthesized yellow powder comprising Type-I and -II HB sheets. White powder comprising Type-III HB sheets was derived by exposing a mixture of Type-I and -II samples to air.

Previous studies using X-ray spectroscopy have focused on examining the electronic structure of 5,7-HB sheets and confirmed gapless states at the Fermi level.^13,15^ However, the expected Fermi edge has not yet been directly observed yet. This fact has left possible existence of the external effects in the sheet, such as presence of synthesis byproducts or impurities in the samples.^13^ Recently, there has been attention on the morphology of atomic sheets that also imposes an additional modification of the electronic state and induces the useful functionality. A notable example is the development of porous boron nitride (BN) nanosheets.^21−23^ The incorporation of Ag nanoparticles into BN sheets effectively modulates pore sizes and enhances absorption capacities.^21^ Furthermore, this nanostructural control method can be used to modify chemical properties, such as catalytic activity and energy storage.^22,23^ Since the 5,7-HB sheets have been synthesized from particles of the mother materials, the reduced spectral weight at the Fermi level is potentially related to the sheet morphology and provides possible regulation of the electronic states through the synthesis. This meets recent demands of diverse applications in materials engineering. It is widely accepted that structural control and alteration are promising strategies for tailoring material properties and exploring new functionalities for our society. Since few efforts have been made to manipulate the properties of HB sheets through morphological control, detailed spectromicroscopic examinations between synthesis and characterization of the 5,7-HB sheet have been highly called for.

In this work, we used microfocused beam photoelectron spectroscopy (PES), Fourier-transform infrared (FTIR) spectroscopy, and scanning electron microscopy (SEM) to systematically investigate the products synthesized at critical steps. The as-obtained Type-I, -II, and -III 5,7-HB sheets possessed an apparent Fermi edge, reduced spectral weight, and an energy gap at the Fermi level, respectively. The semimetallicity of the 5,7-HB sheets was confirmed by directly observing the Fermi edge of the Type-I sample. For the Type-II sample, the spectral suppression was related to terminal B–H bonds at holes in the sheet and likely caused by the localization effect induced by the porous structure that formed during synthesis. The Type-III sample was oxidized to synthesize the energetically gapped or insulating sheet. This research elucidates the origins of the macroscopic data previously reported for 5,7-HB sheets^13,15^ and provides procedures for synthesizing 5,7-HB sheets possessing different structural morphologies and corresponding electronic states. This approach provides an alternative perspective for chemically modifying the electronic states of atomic layers. Our findings contribute insights into understanding the interplay between 2D sheet configurations and electronic states and provide a novel example for developing materials possessing tunable conductivities, bandgaps, and other customizable electronic properties.

Three types of 5,7-HB sheets were obtained at critical stages of the synthesis, as shown in Figure 1b,c. The 5,7-HB sheets were synthesized based on the liquid exfoliation of boron layers in the YCrB_4_ crystal.^13,14,17^ Initially, polycrystalline YCrB_4_ was synthesized and crushed to pieces at a size of tens of micrometers and then mixed with cation exchange resin to initiate the ion exchange, as shown in Figure 1b and Supporting Information S1. Additionally, the identical weight of YCrB_4_ crystals was further ball-milled below 10 μm to facilitate another group of ion exchange, as shown on the right pathway in Figure 1b. Yellow powders comprising Type-I and -II 5,7-HB sheets were synthesized from YCrB_4_ crystals larger and smaller than 10 μm, respectively, as displayed on the left side of Figure 1c. The Type-III sample was prepared by exposing the Type-I and -II samples to an ambient atmosphere until the color changed to white, as shown on the right side of Figure 1c. More details regarding the sample fabrication can be found in Supporting Information S1.

To examine the electronic properties of the 5,7-HB sheets in detail, Figure 2a shows the contributions of the B and H orbitals to the band dispersions of the 5,7-HB structure. More bands appear near *E*_*F*_ for the α_2_ structure than for the α_1_ structure. At the high symmetry points X and Y of the Brillouin zone in the binding energy range 0.2–0.9 eV, degenerate and nondispersing bands appear for the α_2_ structure. These band dispersion curves contribute to characteristic features in the calculated density of states (DOS) (Supporting Information S2, Figure S1a,b). Further analysis *via* partial DOS reveals that these features are predominantly derived from boron orbitals, particularly the in-plane σ band. One can image the electronic state distribution for α_1_ and α_2_ HB sheets from the corresponding spatial distributions for the wave functions, as provided in Supporting Information S2. For both α_1_ and α_2_ HB sheets, the Dirac nodal loops are attributed to the boron orbitals because of the overlap of in-plane (σ) unoccupied and out-of-plane (π) occupied bands. However, the π band is antilocalized in α_2_ HB sheets compared with being localized in α_1_ HB sheets. The gapless electronic states can be attributed to the semimetallicity of the 5,7-HB sheets, as previously discussed.^13,15^ Based on the calculated DOS data (Supporting Information Figure S1), the thermally broadened DOS spectra for α_1_ and α_2_ HB sheets were calculated in Figure 2b. The calculation was performed under the actual measurement temperature and energy resolution. One can observe a clear spectral edge near the Fermi level for the α_2_ type HB sheets, while the intensity at *E*_*F*_ is suppressed for the α_1_ HB sheets. Additionally, several features can be found at 0.2 and 0.6 eV below *E*_*F*_. This characteristic can be experimentally confirmed by using photoemission spectroscopy (PES) to probe the valence bands.

**Figure 2:**
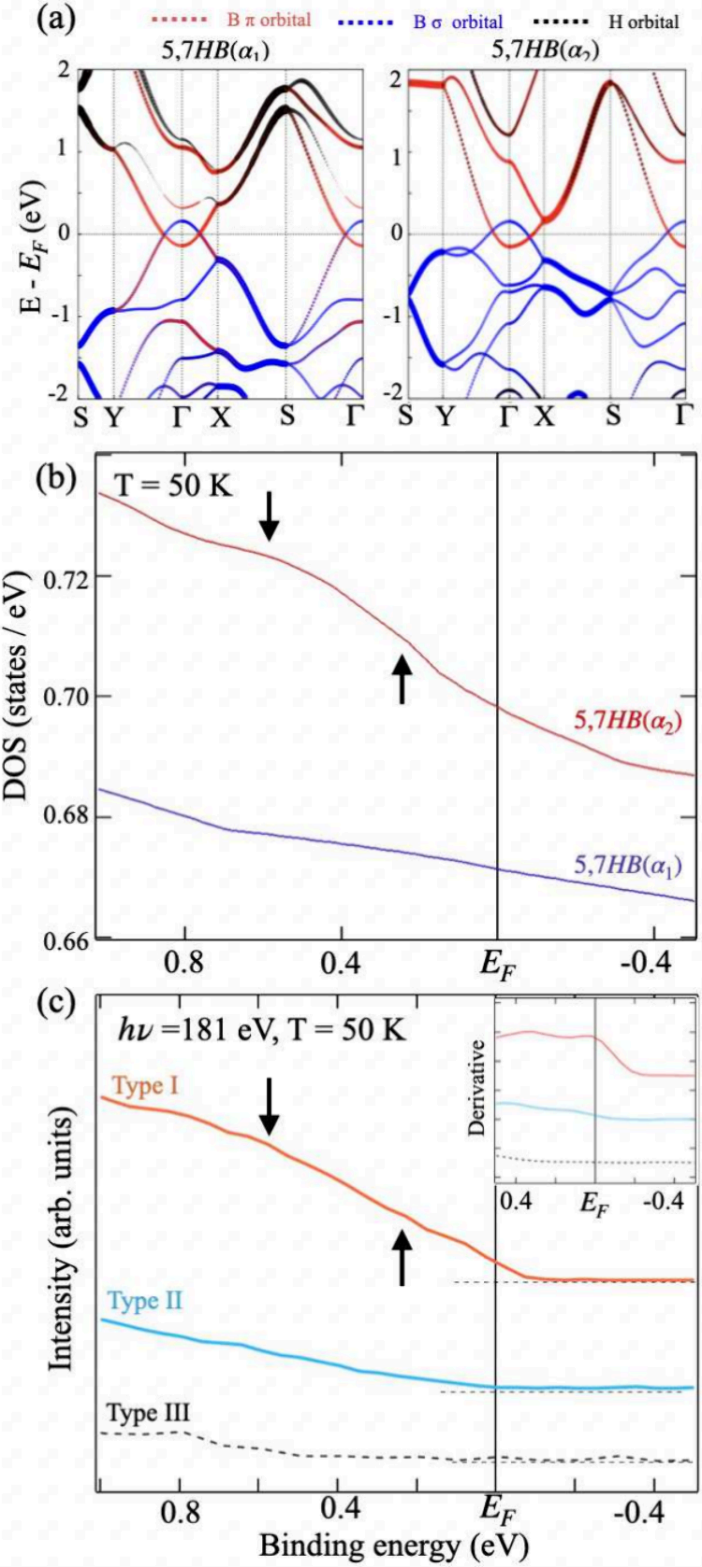
(a) Band dispersions of 5,7-HB sheets (α_1_ and α_2_ type), proportional to projections of B σ (*s* + *p*_*x*_ + *p*_*y*_), B π (*p*_*z*_), and H (*H*_*s*_) orbitals. The intensities of the B and H orbitals are determined by their respective contributions. To enhance the visibility of the H orbitals, their contribution ratio was increased by a factor of 10. (b) Theoretically calculated thermally broadened density of states (DOS) for HB sheets. (c) Energy distribution curves (EDCs) obtained using photon energy *hν* = 181 eV for Type-I, -II, and -III HB sheets (orange, blue, and black dashed curves, respectively). Measurements were conducted at 50 K. The spectra are offset vertically for the sake of clarity. The inset displays the corresponding first derivatives of these curves.

Figure 2c shows the set of PE spectra recorded for the Type-I, Type-II, and Type-III 5,7-HB sheets. In the PE spectrum for the Type-I HB sheets, a Fermi edge appears at *E*_*F*_ and is confirmed by a peak at *E*_*F*_ in the corresponding first derivative spectrum shown in the inset.^24^ However, in the PE spectrum for the Type-II HB sheets, the spectral edge is suppressed and appears as a spectral tail up to *E*_*F*_. The PE spectra of the as-synthesized Type-I and -II powders indicate that the 5,7-HB sheets possess gapless electronic states, as theoretically predicted and reported in previous X-ray spectroscopy studies.^13,15^

A close examination of the PE spectrum for the Type-I powder reveals characteristic features, as indicated by the arrows in Figure 2c, that also appear in the corresponding calculated DOS spectrum in Figure 2b. This spectral similarity suggests that the Type-I HB sheets possess an atomic arrangement of the α_2_ structure. The PE spectrum for the Type-III sheets does not display any material-derived signal at *E*_*F*_, indicating that this sample possesses a gapped or insulating electronic structure. This drastic change was induced by oxidation after exposure to air (Figure 1c). At *E*_*F*_, this divergent electronic behavior suggests that various HB structures could exist, depending on the synthesis conditions, including the mother material’s size and sample’s storage environment.

Next, the chemical properties and origins of the distinctive electronic states of the three types of HB sheets are explored. Core-level PES is a potent method for examining various chemical states. Because the HB sheets comprised microflakes, we used microfocused core-level PES. After the entire sample surface was scanned, the locations of the HB microflakes were identified, as described in Supporting Information S3. Figure 3a shows a collection of B 1s core-level spectra for the three types of HB sheets. In the spectra for the Type-I and -II sheets, peaks *B*_1_ and *B*_1_*′* appeared at binding energies of approximately 187–188 eV, respectively, and were assigned to the negatively charged boron atoms in hydrogen boride (HB), as previously reported.^13−15,17,25^ The absence of Y and Cr which may remain from the mother material YCrB_4_ crystals, was confirmed with a combined measurement from a synchrotron and laboratory X-ray photoelectron spectroscopy (XPS) system (Supporting Information S4). These spectra show the absence of a peak attributed to boron oxide at approximately 192 eV, indicating the high purity of the 5,7-HB sheets. Notably, for both Type-I and Type-II samples, peak shoulders are observed at 186–187 and 189–190 eV. The former can be attributed to B–H bonding,^13,26^ while the latter corresponds to B–B bonding,^26^ which are naturally present in the HB structural network. In the spectrum for the Type-I HB sheets, the B 1s peak at 187.8 eV was shifted toward a binding energy slightly higher than that of the counterpart peak at 187.5 eV in the spectrum for the Type-II HB sheets, indicating different chemical environments in both samples. The spectrum for the Type-III HB sheets is different from those of the Type-I and -II HB sheets. In addition to the B 1s peak at 187.8 eV in the spectra for the 5,7-HB sheets, a prominent peak (*B*_2_) was observed at a binding energy of 192.0 eV and was attributed to oxidized boron compounds, such as B_2_O_3_ or B(OH)_3_.^13^ Consequently, the Type-III sample comprised partially oxidized 5,7-HB sheets.

**Figure 3:**
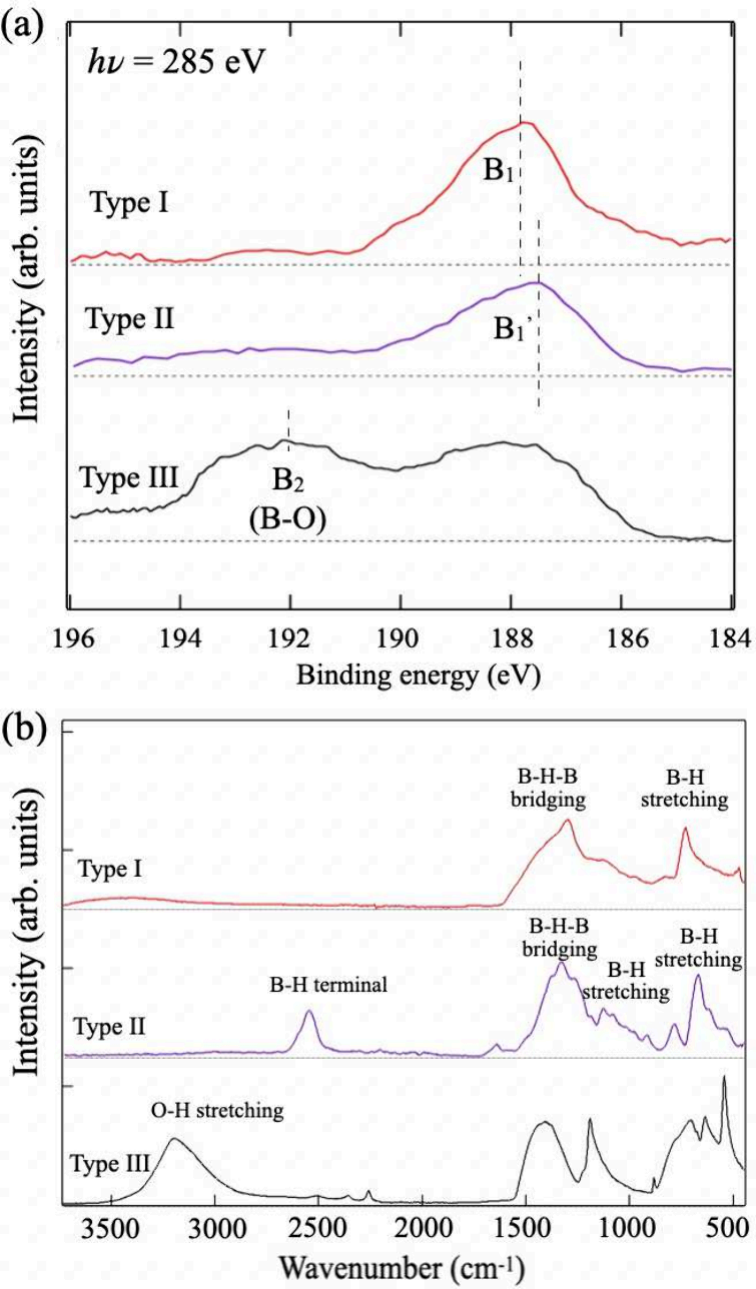
Spectral data for 5,7-HB samples. (a) Collection of B 1s core-level photoemission spectra recorded at *hν* = 285 eV for Type-I, -II, and -III HB samples (indicated by red, purple, and black curves, respectively). (b) FTIR spectra for Type-I, -II, and -III HB samples (indicated by red, purple, and black curves, respectively).

To further characterize the chemical bonds in the as-synthesized HB sheets, Figure 3b shows the FTIR spectra recorded in the range 450–4000 cm^–1^ for the three sample types and encompassing vibrations associated with various B–H bonds. Corresponding FTIR spectra for which absorption peaks were assigned based on previously reported spectral peaks.^13,14,16,17,25,27^ In the spectra for the Type-I and -II HB sheets, vibrational absorption peaks attributed to the bridging and stretching modes of the B–H–B (3c–2e) and B–H (2c–2e) bonds are observed at 1350 and 710 cm^–1^, respectively. In addition, the signals of the B–H stretching mode were identified at approximately 1150 cm^–1^ and were more intense in the spectrum of the Type-II sample than in that of the Type-I sample. Notably, the vibrational peak at 2500 cm^–1^, attributed to the stretching mode of the terminal B-H (2c, 2e) bond, was observed exclusively in the spectrum for the Type-II 5,7-HB sheets. Because terminal B–H bonds are at the edges of the sheets, the Type-II HB sheets are likely to possess more defects, such as holes, than the Type-I HB sheets. For the Type-III HB sheets, the FTIR spectrum appears to be different from those of the Type-I and -II sheets, which is similar to the spectral trends shown in Figure 3a. Notably, a vibrational absorption peak attributed to the O–H stretching mode emerges at 3200 cm^–1^. Because the PE spectrum for the Type-III HB sheets exhibited a peak attributed to oxidized boron, as shown in Figure 3a, the peak attributed to O–H bonds in the corresponding FTIR spectrum indicates the formation of boric acids in the Type-III HB sheets, likely because of oxygen (O_2_) and water molecules (H_2_O) adsorbed on the 5,7-HB sheets during exposure to air (Figure 1).^28,29^

The FTIR spectra of the Type-I and -II HB sheets show apparently different ratios for the numbers of B–H–B (3c–2e) and terminal H–B (2c–2e) bonds in the HB sheets and at the sheet edges, respectively. Because the Type-II HB sheets are defect-rich compared with the Type-I HB sheets, the chemical situation likely perturbs the electrons in the sheets and slightly changes the local bonds, as confirmed by the energy (frequency) shift in the corresponding peaks in the PES (FTIR) spectra. These experimental results for the HB sheets reveal the unique relationship between the spectral appearance of the Fermi edge and the vibrational peak at 2500 cm^–1^. Figure 2b and c shows the apparent Fermi edge that matches with the Type-I HB sheets, while the corresponding FTIR provides negligible B-H vibrational peak. The FTIR signal is assigned to the mode at the terminal H–B bond at the edge of the HB layer, indicating the incompleteness of the sheet.

To investigate the structural morphology, Figure 4 presents scanning electron microscopy (SEM) images of Type-I and Type-II HB sheets. The microflakes in the Type-I HB sheets, as shown in Figures 4a,b, possess flat surfaces and sharp edges, while those in the Type-II HB sheets possess rough surfaces and roundish edges, as shown in Figure 4c,d. A close examination of the Type-II HB sheets reveals that they possess pores of various sizes and exhibit localized layer stacking. The outer edges and holes of the microflakes possess terminal H–B bonds. The microflakes in the Type-II HB sheets possess more holes and, accordingly, more terminal H–B bonds than the microflakes in the Type-I HB sheets [sketched in Figure 4e], which are consistent with the signals in the FTIR spectrum for the Type-II HB sheets, as shown in Figure 3b. Notably, for large holes, the HB sheets possess a ribbon network, as shown in Figure 4f, indicating that either uniform or porous HB sheets can be prepared, depending on the synthesis step. The porous structures were further confirmed using a different approach as follows: The powder comprising the Type-II HB sheets was dissolved in acetonitrile and spin-coated on a Au substrate in a N_2_ environment. The SEM images (Figures 4g,h) depict the morphologies of the Type-II HB sheets, revealing a distinct porous structure. The enlarged holes that formed during spin-coating suggest that the HB sheet structure was likely stretched. Under spin-coating conditions, the morphological variation could reinforce the ribbon network’s structure.

**Figure 4:**
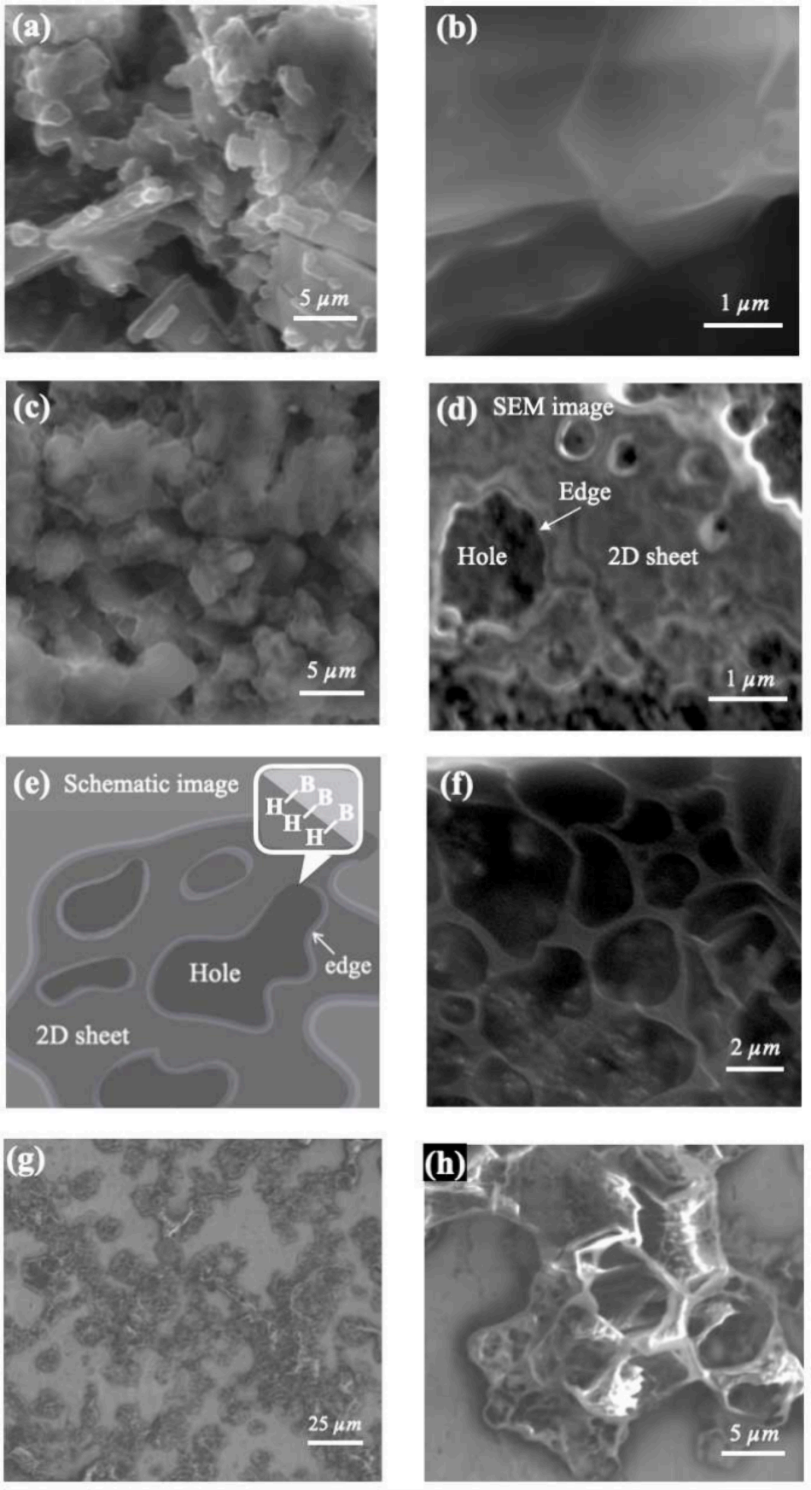
SEM images of powders comprising (a) Type-I and (c) Type-II HB sheets. HB sheets of (b) Type-I and (d) Type-II dispersed in acetonitrile, revealing detailed structures of individual flakes. (e) Schematic of a porous HB sheet. (f) Ribbon network in a Type-II HB sheet. (g,h) SEM images of spin-coated Type-II HB flakes.

According to Figure 2a,b, the Fermi edge for the Type-I HB sheets can be described based on the electronic structure (DOS) of the semimetallicity. However, at the Fermi level, the intensity at the Fermi level for the Type-II HB sheets is weaker than that for the Type-I HB sheets and is likely related to the porous structure (Figure 4d). The region between the holes is restricted to several micrometers and further possesses stacked layers containing various nanostructures, such as approximately 100-nm-wide nanoribbons. Because the Fermi wavelengths of the α_1_ and α_2_ HB sheets are 7.52 and 7.74 nm (according to the calculated bands in Figure 2c), respectively, the electronic states are likely modified by a possible size effect. When an electron is confined in a region for which the width is on the order of the electron’s wavelength, the energy levels are quantized (through the quantum confinement effect) and become energetically continuous with increasing regional width, forming an energy band. Because the width of the nanoribbons in the porous HB sheets is on the order of the electron’s wavelength, the electronic states of the Type-II HB sheets are likely localized as compared with those of the Type-I wide-area HB sheets. This localization model describes the appearance of the Fermi edge in the PES for two types of HB sheets (Figure 2c). Thus, by modifying the chemical reaction parameters during synthesis, the structural morphology of the HB sheets can be changed, and consequently, the electronic states can be regulated *via* the quantum size effect.

In summary, we have controllably synthesized HB sheets that possess either an apparent Fermi edge, suppressed spectral weight, or a Fermi-level energy gap, as probed using microfocused beam PES. In the Type-I HB sheets, the gapless electronic state is consistent with the semimetallicity of the HB sheet, as expected based on first principles calculations. In the Type-II HB sheets, the porous structure reduced the DOS at the Fermi level, as made evident by SEM images and FTIR spectra. The Type-III insulating HB sheets were fabricated by oxidizing the HB layers. This study not only elucidates the electronic states in microflakes but also uncovers the intricate interplay among the chemical bonding, electronic states, and material morphology in the HB sheets. The discovery of different HB sheet types and their distinct electronic properties provides a promising approach for regulating the metallicity of semimetals by precisely tuning their chemical structures and underscores the vast potential of HB sheets for diverse applications in nanoelectronics and beyond.

## Experimental Methods

### Microfocus PES

Examinations of electronic states of the HB samples were made by photoemission spectroscopy (PES) at the synchrotron radiation beamline BL–28A in the Photon Factory at the High Energy Accelerator Research Organization (KEK).^30^ The beamline provided the microfocused X-ray beam with a spot size of 30 μm (horizontal) × 15 μm (vertical). The HB powders were dispersed on a Au substrate, and the surface was cleaved with a Kapton tape before being installed into the UHV chamber for PES measurements. Locations of the HB microflakes were determined by 2-D scanning of the photoemission signal at the B 1s core-level over the sample. PE spectra and the corresponding EDCs were recorded by integrating the whole region of the first Brillouin zone in k-space at 50 K with the instrumental energy resolution of 50 meV.

### FTIR Measurement

Chemical bonding configurations were investigated by probing the vibrational modes *via* FTIR spectroscopy. The FTIR spectra were recorded with a commercial system, ALPHA II (BRUKER). The HB powder samples were dispersed on the prism holder and measured using the attenuated total reflectance (ATR) method under an Ar gas environment at room temperature.

### SEM Measurement

Structural morphology of the HB microflakes was studied by scanning electron microscopy (SEM, JEOL JXA-8530F). The experiment was performed at room temperature.

### First-Principles Calculations

The electronic structure of HB sheets was calculated using density functional theory (DFT) with the Quantum ESPRESSO code.^31−33^ Ultrasoft pseudopotentials were employed to describe the electron-ion interaction.^34^ Spin–orbit coupling is neglected and for the exchange-correlation term, the generalized gradient approximation (GGA) with nonrelativistic Perdew-Burke-Ernzerhof parametrization is used.^35^ Valence wave functions were expanded using a planewave basis, and the cutoff energies were set at 80 and 480 Ry for wave functions and charge density, respectively. The k-point grid on the Brillouin zone was taken at 16 × 8 × 1 in the band calculations. For DOS calculation, the Gaussian broadening width is set as 0.01 Ry and the *k*-point grid on the Brillouin zone is taken as 32 × 16 × 1. The thermally broadened DOS was calculated with the temperature *T* = 50 K. The lattice parameters of the HB sheet were adopted from a previous report as *a* = 5.875 Å, *b* = 11.292 Å for the α_1_ and α_2_ HB.^12,13^ To simulate isolated HB monolayers, the sheet was separated by periodic images with a vacuum region of 15 Å.

## Supporting Information Available

Details about the synthesis, sample preparation, spatial distribution of the wave function, X-ray photoemission spectra for Y 3d and Cr 2p, and scanning method for locating microflakes (PDF)
