# Supplementary material for: Fermi Edge of Semimetallic Borophane Sheets and its Reduction by a Porous Structure
Source: J Phys Chem Lett. 2024 Sep 6;15(37):9349–55. doi: 10.1021/acs.jpclett.4c01869 (PMC11418824; doi:10.1021/acs.jpclett.4c01869)
Supplement: Supplementary file 1 — jz4c01869_si_001.pdf [file jz4c01869_si_001.pdf]

Supporting Information: Fermi Edge of Semimetallic Borophane Sheets and its Reduction by a Porous Structure

Xiaoni Zhang,[†] Masashige Miyamoto,[†] Mei Yuan,[‡] Yuki Tsujikawa,[†] Kazuki Yamaguchi,[†] Masafumi Horio,[†] Kenichi Ozawa,[¶] Kunio Yubuta,[§] Takahiro Kondo,[‡] and Iwao Matsuda*,[†]

†*Institute for Solid State Physics (ISSP), The University of Tokyo, Kashiwa, Chiba 277-8581, Japan*

‡*Institute of Pure and Applied Sciences, University of Tsukuba, Tsukuba, Ibaraki 305-8573, Japan*

¶*Institute of Materials Structure Science, KEK, Ibaraki, Japan*

§*Faculty of Engineering, Kyushu University, Fukuoka, Fukuoka 819-0395, Japan*

E-mail: imatsuda@issp.u-tokyo.ac.jp

S1. Materials synthesis and Sample preparation

Synthesis of three types of the 5,7-HB sheet: Three types of the HB samples were prepared by the ion exchange reaction. Initially, the mother material, polycrystals of $YCrB_4$ were prepared using the melting method, with a composition ratio of Y: Cr: B = 1: 1: 4. For one group of ion exchange reaction, 370 mg of $YCrB_4$ crystals were continuously stirred with 30 ml cation ion exchange resin beads (0.5-1 mm, Amberlite IR120B H HG, Organo Corp., Tokyo, Japan) in 200 ml acetonitrile solvent (99.5%, JIS special grade, FUJIFILM

Wako Pure Chemical Corp, Osaka, Japan) under an inert Ar gas environment at room temperature for three days. To enhance the reaction, 1 ml of 1 M hydrochloric acid (HCl) was added for every 2 mmol of $YCrB_4$. Notably, the ion exchange occurs at the liquid/solid interface of the crystal, resulting in distribution of the HB sheets in the solvent. Powders of the Type-I and Type-II HB sheets were obtained through filtration and the subsequent concentration. The as-synthesized powders (Type-I and Type-II) were exposed in ambient atmosphere at room temperature for 3 hours to prepare the Type-III sample. After the treatment, all the synthesized HB samples were stored in a glove box, filled with Ar gas, to minimize the further contamination.

Sample preparation for Micro-PES and XPS measurement: A 5 mg powder of 5,7-HB sheets was dispersed onto a polycrystalline Au substrate. The Au surface with the powder sample was then covered with Kapton tape. The entire preparation process took place within an Ar environment inside a glove box. Subsequently, the prepared samples of three different types were transferred to the synchrotron radiation beamline BL–28A using a vacuum suitcase. To prevent contamination during transfer, the samples were moved into the load lock chamber via a glove bag filled with N_2 gas. The cleavage of the samples was carried out by removing the Kapton tape inside the glove bag.

Sample preparation for microscopy observation: The HB sheet powder was dispersed onto carbon tape and cleaved by Kapton tape, then installed in the SEM chamber under atmospheric conditions. For detailed observation of the HB flake edges, 10 mg of the powder was dissolved in 3 ml of acetonitrile and dropped onto copper grids. The grids were then mounted onto the sample holder for electron microscopy inside a glove box. The prepared sample with the holder was subsequently installed in the SEM chamber under atmospheric condition.

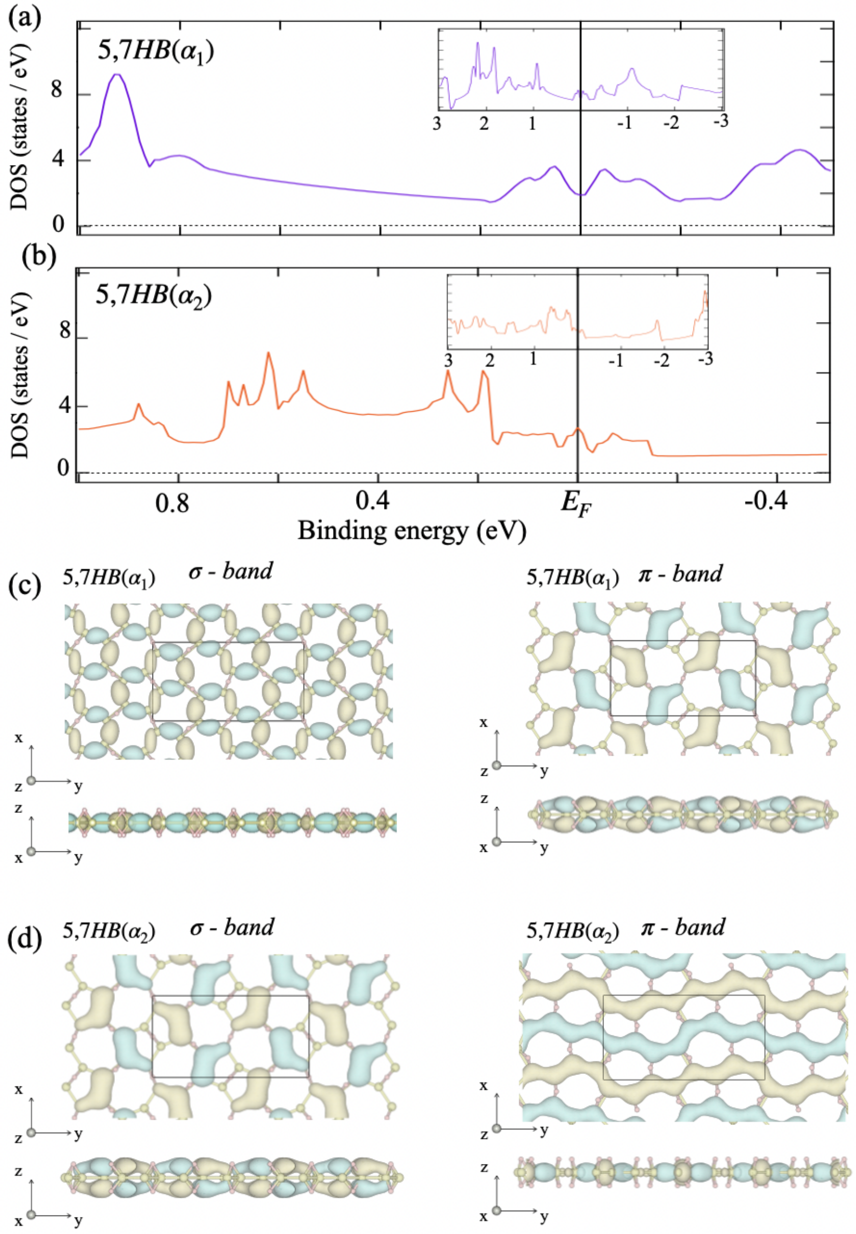

Figure S1. Density of states (DOS) calculation for (a) 5,7-HB (α_1) and (b) 5,7-HB (α_2). Spatial distributions of wave functions for the unoccupied σ band (left) and occupied π band (right) of 5,7-HB sheets (c) α_1 and (d) α_2 structure.

S2. Spatial distributions of wave functions of 5,7-HB sheets

The theoretically predicted density of states (DOS) of the α_1 and α_2 structures are shown in Fig. S1(a) and (b), respectively. These results, as shown in the corresponding insets, are consistent with previously reported results.[1] At the Fermi level (E_F), the spectra calculated for both the α_1 and α_2 structures reveal gapless DOS features. Notably, the spectrum for the α_2 structure exhibits characteristic features in the binding energy range 0.2–0.9 eV while the spectrum for the α_1 structure shows a featureless DOS in the same region. These DOS features were verified through electronic band calculations in Fig. 2(a) and EDC in Fig. 2(c) in the main text. The spatial distributions of wave functions at E_F for the σ and π bands of the two structures of 5,7-HB are shown in Fig. S1(c) and (d).

S3. Location of single flake HB by Micro-focused PES

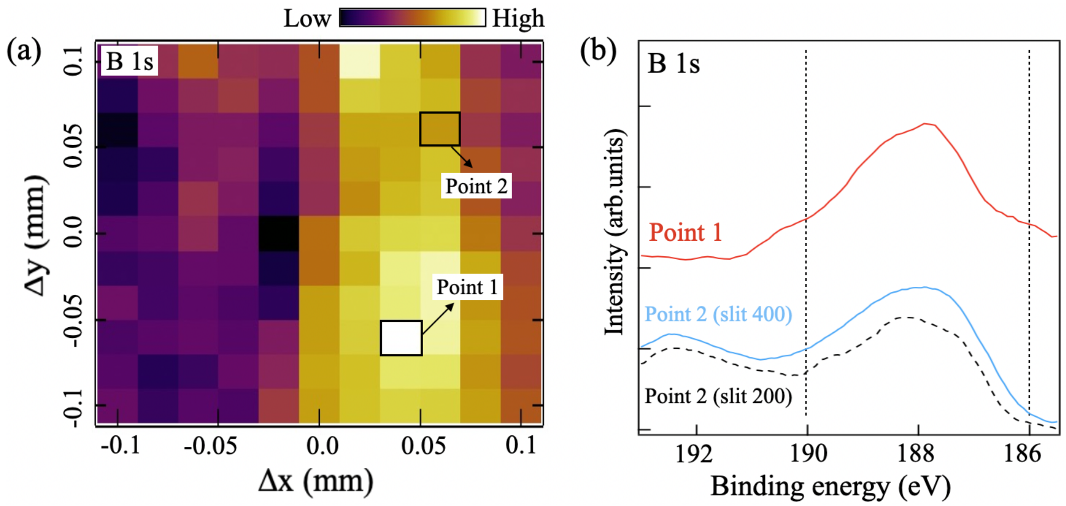

Figure S2. (a) Spatial mapping of photoemission intensity integrated over the B 1s core-level (E_B range of 186–190 eV), measured with $h\nu = 285$ eV. The step size was 200 µm along the x and y directions. (b) Intensity spectra for the B 1s core level measured at Point 1 (red curve) and Point 2 (blue curve). The absence of a charging effect was confirmed (black dashed line). All measurements were performed at 50 K.

To identify individual 5,7-HB flakes from the substrate and other impurities, including boric acid, we performed scanning micro-photoelectron spectroscopy (micro-PES) measure-

ments with 20 μm step size. The spatial distribution of photoelectron intensity integrated over a binding energy (E_B) range of 186-190 eV was mapped using a photon energy $h\nu =$ 285 eV. An illustrative example of this spatial mapping for type-I HB sheets is presented in Fig. S2(a). Regions of high intensity, depicted in yellow-to-white colors, likely correspond to the HB sheets, characterized by a negatively charged B 1s core-level peak, as shown in Fig. S2(b). Specifically, at a marked position labeled as Point 1, a B 1s peak appeared at 187.8 eV (represented by the red curve in Fig. S2(b)), indicating the presence of pure HB sheet without oxide contamination. In contrast, at Point 2, the boron oxide peak at 192.5 eV contributed visible intensity (shown by the blue curve in Fig. S2(b)), signifying oxide contamination and lowering B 1s intensity.

Given the insulating nature of boron oxide components, Point 2 might have experienced charge-up, potentially affecting spectroscopic accuracy. To validate this, we repeated the measurement at Point 2 using a narrower slit (resulting in lower photon electron flux), as indicated by the black dashed curve in Fig. S2(b). The negligible shift in the B 1s peak confirmed that there was no charge-up effect on our measurement spectrum.

Adopting the sample location method described above, we mapped and located the single flake of three types of HB samples for the micro-PES and XPS measurement.

S4. Confirmation of absence of Y and Cr

Due to the limited photon energy available in the synchrotron radiation beamline BL-28A (up to 300 eV), measurements of Cr 2p peak were not feasible. Therefore, we conducted measurements on the Type-I, -II, and -III samples using a laboratory X-ray photoelectron spectroscopy (XPS) system. The XPS measurements were performed with an incident X-ray beam energy of hν = 1253.6 eV (Mg Kα) using a Scienta Omicron DA30-L-8000 photoelectron analyzer.

Figures S4-S6 present the core-level spectra (B 1s, Y 3d, and Cr 2p) obtained from the

three sample types using the X-ray tube. Additionally, the B 1s and Y 3d spectra obtained at the synchrotron facility are included in the figures. The absence of Y and Cr signals was confirmed, and it was determined that the (reduced) Fermi-edge is not due to any remnant $YCrB_4$ crystal contributing spectral weight near the Fermi level.

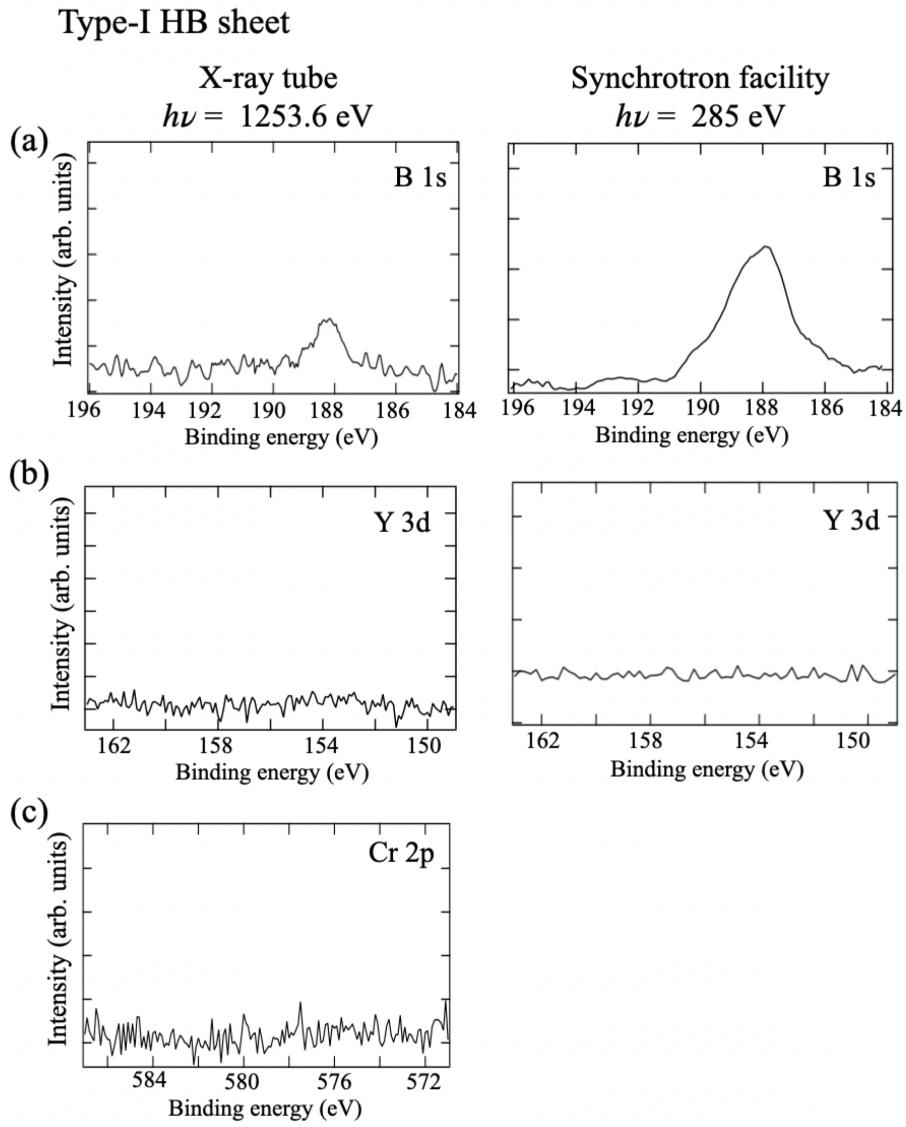

Figure S3. Collection of XPS spectra for Type-I HB sample at the binding energy regions of (a) B 1s, (b) Y 3d and (c) Cr 2p, taken at laboratory XPS (left) and synchrotron facility (right), respectively.

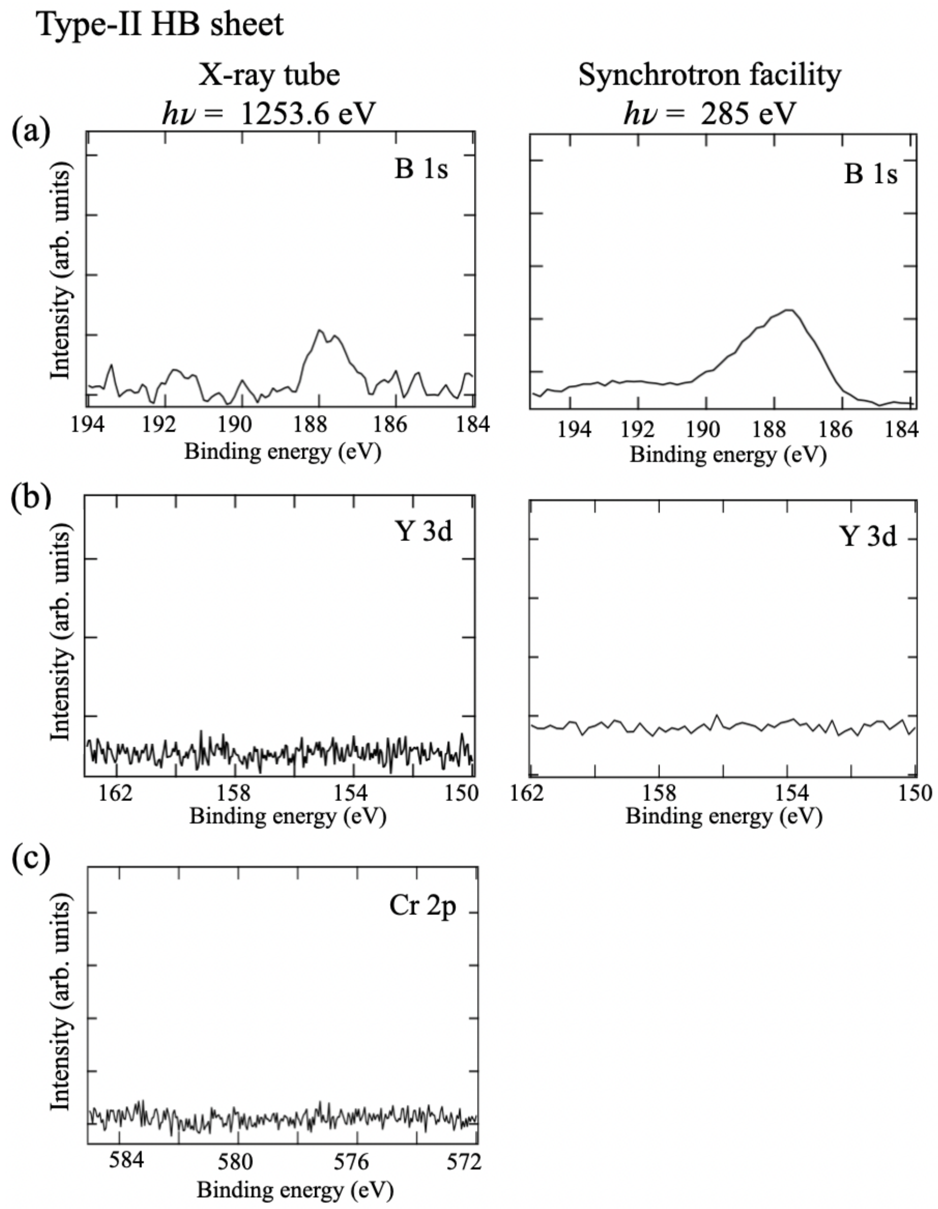

Figure S4. Collection of XPS spectra for Type-II HB sample at the binding energy regions of (a) B 1s, (b) Y 3d and (c) Cr 2p, taken at laboratory XPS (left) and synchrotron facility (right), respectively.

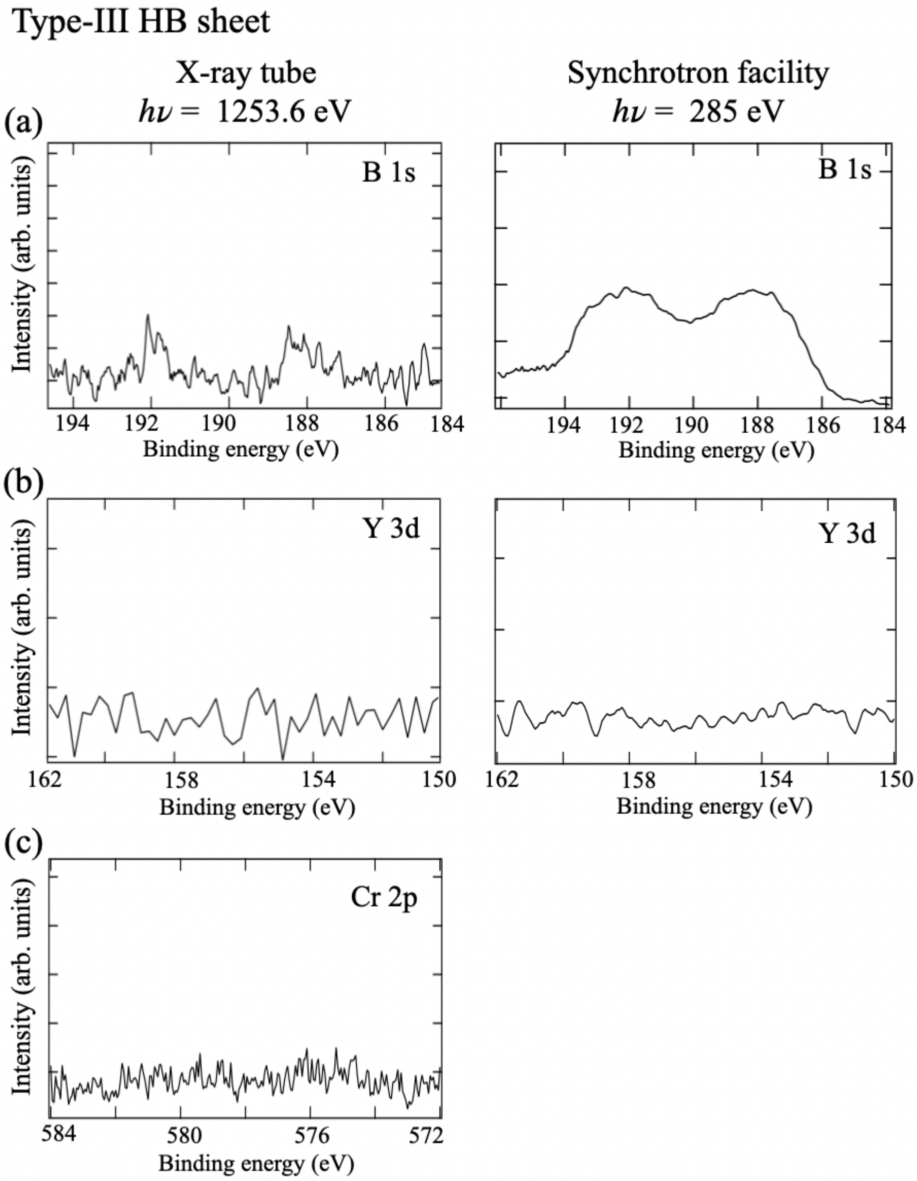

Figure S5. Collection of XPS spectra for Type-III HB sample at the binding energy regions of (a) B 1s, (b) Y 3d and (c) Cr 2p, taken at laboratory XPS (left) and synchrotron facility (right), respectively.

References

(1) Niibe, M.; Cameau, M.; Cuong, N. T.; Sunday, O. I.; Zhang, X.; Tsujikawa, Y.; Okada, S.; Yubuta, K.; Kondo, T.; Matsuda, I. Electronic structure of a borophene layer in rare-earth aluminum/chromium boride and its hydrogenated derivative borophane. *Phys. Rev. Mater.* **2021**, *5*, 084007.
